# Microgravity Promotes Differentiation and Meiotic Entry of Postnatal Mouse Male Germ Cells

**DOI:** 10.1371/journal.pone.0009064

**Published:** 2010-02-04

**Authors:** Manuela Pellegrini, Sara Di Siena, Giuseppina Claps, Silvia Di Cesare, Susanna Dolci, Pellegrino Rossi, Raffaele Geremia, Paola Grimaldi

**Affiliations:** Department of Public Health and Cellular Biology, University of Rome Tor Vergata, Rome, Italy; City of Hope National Medical Center, United States of America

## Abstract

A critical step of spermatogenesis is the entry of mitotic spermatogonia into meiosis. Progresses on these topics are hampered by the lack of an *in vitro* culture system allowing mouse spermatogonia differentiation and entry into meiosis. Previous studies have shown that mouse pachytene spermatocytes cultured in simulated microgravity (SM) undergo a spontaneous meiotic progression. Here we report that mouse mitotic spermatogonia cultured under SM with a rotary cell culture system (RCCS) enter into meiosis in the absence of any added exogenous factor or contact with somatic cells. We found that isolated Kit-positive spermatogonia under the RCCS condition enter into the prophase of the first meiotic division (leptotene stage), as monitored by chromosomal organization of the synaptonemal complex 3 protein (Scp3) and up-regulation of several pro-meiotic genes. SM was found to activate the phosphatidyl inositol 3 kinase (PI3K) pathway and to induce in Kit-positive spermatogonia the last round of DNA replication, typical of the preleptotene stage. A PI3K inhibitor abolished Scp3 induction and meiotic entry stimulated by RCCS conditions. A positive effect of SM on germ cell differentiation was also observed in undifferentiated (Kit-negative) spermatogonia, in which RCCS conditions stimulate the expression of Kit and Stra8. In conclusion, SM is an artificial environmental condition which promotes postnatal male germ cell differentiation and might provide a tool to study the molecular mechanisms underlying the switch from mitosis to meiosis in mammals.

## Introduction

In the adult mouse testis, spermatogenesis originates from spermatogonial stem cells (Asingle, As), that can self-renew or differentiate into committed paired (Ap) and aligned (Aal) spermatogonia. The heterogeneous population of germ cells, including stem cells and committed spermatogonia, is collectively called undifferentiated spermatogonial population, or Kit-negative spermatogonia. This population expresses different stem cell markers (such as Plzf, Oct4, Nanos3) but does not express the Kit tyrosine-kinase receptor [1]–[4]. Approximately 6 days after birth Aal cells begin to differentiate into A1 to A4, Intermediate, B spermatogonia, and finally preleptotene spermatocytes which undergo meiosis [4]. The appearance of A1 coincides with the expression of the Kit receptor, a marker of differentiating spermatogonia that is expressed until the preleptotene stage and then is down-regulated at the time of meiotic entry [5]–[7]. The cell population from type A1 to type B spermatogonia is called differentiating (Kit-positive) spermatogonia, which, after a defined number of cell divisions, enter into the meiotic program. Up to now, only two agents have been postulated to have a role in the induction of meiotic entry in male mitotic germ cells: all-trans retinoic acid (ATRA) and Kit Ligand (KL). ATRA has been shown to determine entry into meiosis of germ cells in the ovary, while, in the fetal testis, the presence of the retinoid-degrading enzyme CYP26B1 prevented its action [8]–[9]. Moreover, we recently demonstrated that, in postnatal testis, ATRA increases meiotic entry of differentiating spermatogonia *in vitro* by activating the Kit signalling pathway [10] and by stimulating a significant increase of Stra8, a fundamental regulator of meiosis in both female and male mice [11]–[12]. Similarly to ATRA, addition of KL, a growth factor essential for survival and proliferation of Kit positive germ cells [5], [13]–[14], increases the percentage of meiotic nuclei in cultured spermatogonia, concomitantly with an up-regulation of Stra8 [10].

We were interested in establishing culture conditions that would eventually allow a spontaneous differentiation of mitotic germ cells toward the meiotic program, in the absence of exogenously added growth factors or contact with supporting somatic cells. Such conditions would be helpful to facilitate studies on the molecular mechanisms that regulate the mitotic-meiotic switch in mammalian germ cells. Recently it has been demonstrated that simulated microgravity (SM) exerts a positive effect on cell proliferation and differentiation in cell types such as periodontal stem cells [15] or osteoclasts and their precursors [16]. As for male germ cells, Di Agostino and co-workers [17] have shown that isolated mouse pachytene spermatocytes cultured under SM undergo spontaneous meiotic progression. SM was also found to increase the number of duplicating germ cells in organ cultures of testicular fragments [18].

Here we report that rotary cell culture system (RCCS) conditions provoke a dramatic increase in the number of meiotic figures in cultured Kit-positive spermatogonia, as revealed by the chromosomal organization of the synaptonemal complex protein 3 (Scp3). We also observed a concomitant increase in the expression of pre-meiotic proteins, such as Kit and Stra8, and of meiotic markers, such as Spo11 and Scp1. We found that SM stimulates the last round of premeiotic DNA synthesis and activates the phosphatidyl inositol 3 kinase (PI3K) signalling pathway in these cells. PI3K activation appears to be required for stimulation of meiotic entry. Moreover SM was also found to promote differentation of Kit-negative spermatogonia.

These data suggest that microgravity elicits spontaneous differentiation of spermatogonia and their entry into the prophase of the first meiotic division.

## Results

### Isolation and Characterization of Kit-Positive Spermatogonia

**Figure 1:**
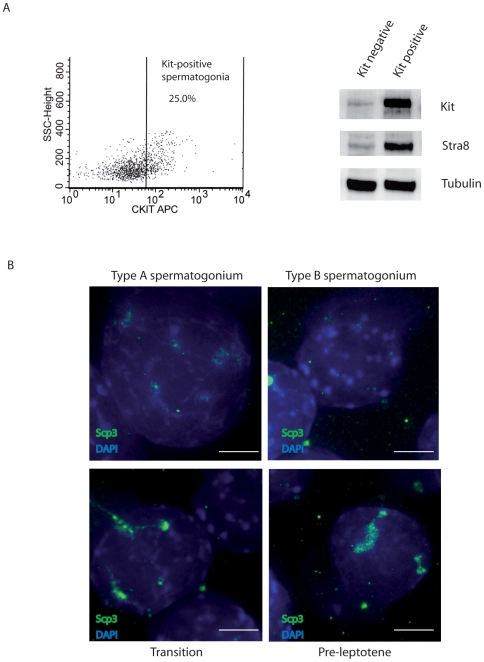
Isolation and characterization of Kit-positive Spermatogonia. A) Germ cells were obtained with sequential enzymatic digestions from testis of 7dpp animals and analyzed by FACS analysis for Kit expression (left panel). Kit-positive spermatogonia were purified using CD-117 conjugated beads, and Kit and Stra8 protein expression were detected by Western blot analysis. Tubulin was used as loading control (right panel). B) Representative images of DAPI (blue) and Scp3 (green) staining of nuclear spreads from isolated Kit-positive spermatogonia. Scp3-negative nuclei with decondensed chromatin (type A spermatogonia) or with heterochromatic nuclei (type B spermatogonia), and Scp3-positive nuclei with small and diffuse spots (Transition Spermatogonia) or with large and defined spots (Preleptotene) are shown. Scale bar, 10 µm.

Spermatogonial germ cell suspensions, including undifferentiated Kit-negative and differentiating Kit-positive spermatogonia, were obtained from testes of immature 7 day post-partum (dpp) mice. To allow the adhesion and removal of most contaminating somatic testicular cells, suspensions were subjected to a pre-plating treatment with fetal calf serum. Kit-positive spermatogonia, were then purified from the germ cell population using anti-CD117 (Kit) conjugated microbeads. We estimated that this class of cells represents approximately 20%–25% of total germ cells. This data was confirmed by cytofluorimetric analysis (Fig. 1A, left panel). The enrichment of the isolated Kit-positive population was tested by western blotting analysis, which showed that Kit and Stra8 are strongly expressed compared to the Kit-negative population (Fig. 1A, right panel). The purity of the Kit positive germ cells, selected by immunomagnetic beads, was routinely confirmed by negativity for the expression, at both protein and mRNA levels, of several markers of undifferentiated germ cells, such as Oct4, Plzf, Nanos3, Bcl6b, and for positivity, besides Kit and Stra8, also for Sohlh1 expression, as previously reported [2 and our unpublished data].

To morphologically characterize the Kit-positive spermatogonia, nuclear spreads were prepared and stained with DAPI and with an anti-Scp3 antibody that detects premeiotic and meiotic nuclei. Based on their nuclear size and morphology [19], [10], several types of spermatogonia were identified in the freshly isolated Kit-positive population (Fig. 1B). Most of spermatogonia nuclei were Scp3-negative and could be identified as type A and type B spermatogonia. Type A showed more homogeneously distributed and less condensed chromatin, while type B possess a typically round nucleus, which is smaller in size and contains several condensed chromatin patches.

A lower percentage of cells were Scp3-positive and could be identified either as preleptotene spermatocytes (2%±0.3% of total Kit-positive germ cells) showing typical Scp3 spots within their nuclei, or as “transition” germ cells (14.5%±1.5%) representing a subclass of type B spermatogonia characterized by few, small and diffuse Scp3 spots, in which nuclear Scp3 protein is already detectable but not yet organized. Notably no meiotic leptotene spermatocytes were found in the Kit-positive population, as monitored by DAPI staining and Scp3 organization.

### SM Condition Increases Meiotic Entry of Kit-Positive Spermatogonia

**Figure 2:**
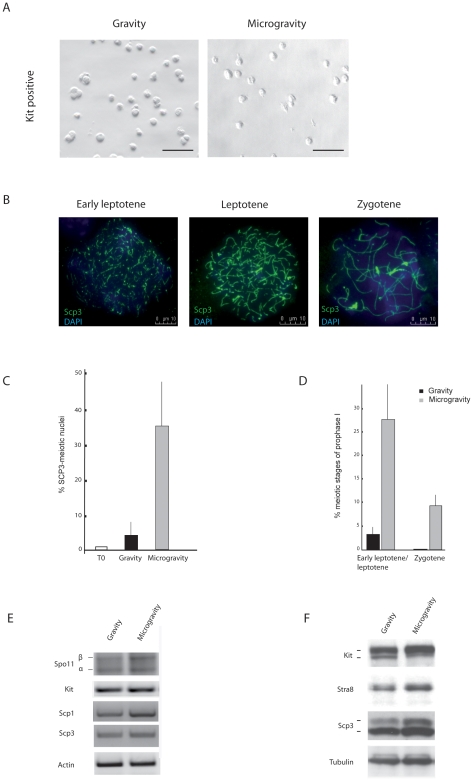
SM induces meiotic entry of Kit-positive spermatogonia. A) Morphology of Kit-positive spermatogonia from testes of 7dpp mice after 2 days of culture under SM and unit gravity seen at light microscopy. Scale bar, 100 µm. B) Representative immunofluorescence images showing Scp3 organization on nuclear spreads at different stages of meiotic prophase I made on cultured spermatogonia. Scale bar, 10 µm. C, D) Histograms representing the percentage of nuclei with a typical meiotic Scp3 organization (C) and relative amount of early leptotene/leptotene and zygotene meiotic figures (D) after 48 h of cultures kept at unit gravity and in SM. The percentage of Scp3-positive cells present in freshly collected Kit-positive cells is shown as T0. The values were obtained by counting 400 nuclei in each sample. Data are means ± SD of at least four independent experiments. P≤0.005. E, F) Representative semiquantitative RT-PCR (E) and western blots (F) for pre-meiotic markers Kit and Stra8 and meiotic markers Spo11, Scp3, Scp1 in spermatogonia under SM versus gravity cultures. Similar results were obtained in three separate experiments. See Table 1 for quantitative data.

SM has been previously reported to influence chromatin condensation and meiotic progression of mouse spermatocytes from the pachytene stage towards the first meiotic division [17]. We decided to test whether the differentiative effect of SM was also exerted on the earlier mitotic stages of spermatogenesis, and, in particular, in Kit-positive spermatogonia, the germ cells prone to enter into the meiotic program. Isolated Kit-positive spermatogonia were cultured for 48 h in RCCS and their survival was monitored by Trypan blue staining and by TUNEL analysis. On the contrary of what previously reported for several other cell types [20]–[21], SM did not affect germ cell survival, since the cell viability rate after 48 h of culture was about 60–70% both in culture at unit gravity and under the RCCS condition (data not shown). Figure 2A shows that Kit-positive spermatogonia cultured for 48 h did not form aggregates when kept in RCCS, a phenomenon that is instead frequently observed when culturing other cell types under SM [22]–[23]. After the 48 h culture period, nuclear spreads were made and stained with Scp3 to detect meiotic stages, as represented in Fig. 2B. Figure 2C shows that the cell population cultured under SM underwent a dramatic increase (10 fold) in the percentage of nuclei with typical meiotic organization of Scp3 onto chromosomes (35.7%±14.3) with respect to cells left at unit gravity (3.60%±2.00). This increase was mainly due to cells at the leptotene stage (27.31%±8.6 with respect to 3.0%±1.5 at unit gravity) (Fig. 2D). We also observed the appearance of cells at the zygotene stage in the SM condition (8.4%±2.1), which were never seen in the culture at unit gravity (Fig. 2D). In line with these results, analysis of nuclear spreads performed after a shorter period of culture under SM (24 h) showed a consistent increase in earlier meiotic stages, mainly in preleptotene/early leptotene nuclear figures (34.5%±7 in SM vs 12.5%±4 at unit gravity; see Fig. 1B and Fig. 2B for representative cell morphology).

These observations were confirmed by transcriptional and translational analysis of pre-meiotic and meiotic markers after 24 h of culture. Semiquantitative RT-PCR analysis of Scp1, the major component of the central region of the synaptonemal complex [24] and Spo11, a type II topoisomerase, needed for meiotic recombination [25], showed an increase of about 2 fold (Fig. 2E; Table 1). A slight increase in the RNA levels of Scp3, but not of Kit, was also observed (Fig. 2E; Table 1). Moreover, western blot analysis of spermatogonia protein extracts showed an increase of about 2 fold for Kit, Scp3 and Stra8 with respect to extracts from cells cultured at unit gravity (Fig. 2F; Table 1). These results indicate that the SM condition favours meiotic progression of the immunomagnetic purified Kit positive spermatogonia, evaluated both at morphological and molecular levels.

**Table 1:** Up-regulation of pro-meiotic markers in Kit-positive spermatogonia from 7dpp testes, cultured under SM with respect to cells kept at unit gravity.

Gene name	mRNA fold induction (RT-PCR)	Protein fold induction (Western blot)
Spo11	1.8±0.3	N.D.
Kit	1.0±0.1	1.8±0.3
Scp1	1.9±0.2	N.D.
Scp3	1.4±0.1	1.9±0.2
Stra8	N.D.	2.2±0.2

### SM Promotes Differentiation of Kit-Negative Spermatogonia

**Figure 3:**
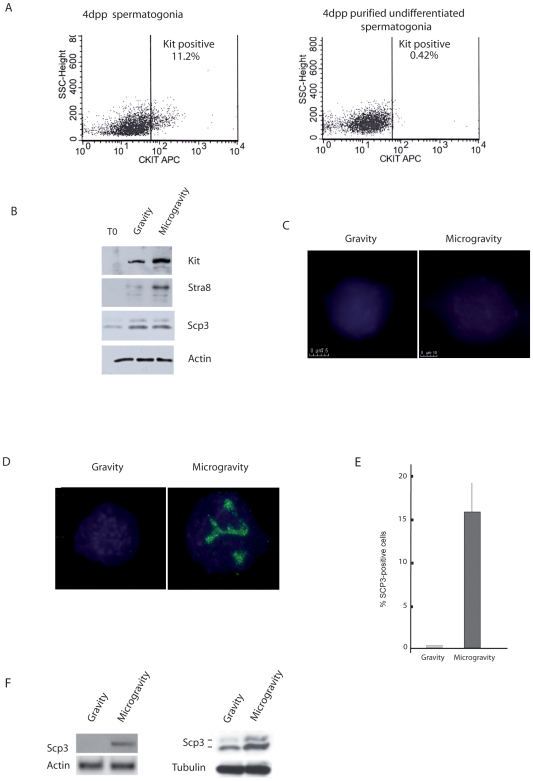
SM accelerates differentiation of undifferentiated Kit-negative spermatogonia. A) FACS analysis of total germ cells population from testes of 4dpp mice before (left panel) and after (right panel) removal of Kit positive cells. B) Western blot analysis of Kit, Stra8 and Scp3 expression in undifferentiated Kit negative cells at T0 and after 24 h of culture under gravity or SM. C) Lack of Scp3 organization on nuclear spreads of undifferentiated spermatogonia after 24 h of culture under either unit gravity or SM conditions. D) Scp3 organization on nuclear spreads of total spermatogonia from 4dpp mice, cultured for 24 h under SM or gravity conditions. Preleptotene-like cells are evident only in microgravity samples. E) Histogram representing the percentage of preleptotene-like nuclei in gravity and SM cultures. The values were obtained by counting 300 nuclei in each sample. Data are means ± SD of at least three independent determinations. P≤0.05. F) Representative semiquantitative RT-PCR (left panel) and western blot (right panel) for Scp3 expression in total spermatogonia from 4pp testes, cultured under SM compared to control cells. Similar results were obtained in three separate experiments.

We investigated whether SM had effects also on the progression of mitotic germ cells at earlier stages of differentiation. Undifferentiated Kit-negative spermatogonia obtained from testes of 4dpp mice, were purified by depletion of Kit-positive cells with immunomagnetic beads and their purity was monitored by FACS analysis (Fig. 3A). The undifferentiated germ cell population was cultured in RCCS or at unit gravity. After 24 h of culture, germ cells were analyzed for the expression of protein markers, important for spermatogonia differentiation and commitment towards meiosis (Kit, Stra8 and Scp3). Interestingly, we observed a strong increase in protein levels of Kit and Stra8 markers in SM conditions, whereas Scp3 levels did not change (Fig. 3B). No Scp3 nuclear organization was evident either in gravity or in SM condition after 24 h of culture (Fig. 3C). These results suggest that microgravity accelerates the *in vitro* spontaneous differentiation of undifferentiated spermatogonia, but it does not induce premature entry into meiosis.

On the other hand, when we cultured total mitotic germ cells isolated from 4dpp testes, prior of immunomagnetic removal of Kit positive spermatogonia, we could appreciate the appearance of a consistent number of Scp3-positive nuclei under SM condition (16%±3.0), while germ cells at unit gravity were completely Scp3-negative (Fig. 3D, E). The Scp3 positive nuclei had the characteristics of a preleptotene-like stage and we never observed early leptotene/leptotene meiotic nuclei, even after more prolonged incubation times. Scp3 espression at both mRNA and protein levels was clearly increased in the total population of 4dpp germ cells cultured for 24 h under the RCCS condition with respect to unit gravity control. (Fig. 3F).

### SM Induces DNA Synthesis in Preleptotene Spermatocytes and Activates the PI3K Signalling Pathway

Since Kit-positive spermatogonia undergo a series of mitotic cell cycles that are required for their subsequent entry into meiosis [10], [26], we investigated whether SM had a positive effect on their proliferation.

**Figure 4:**
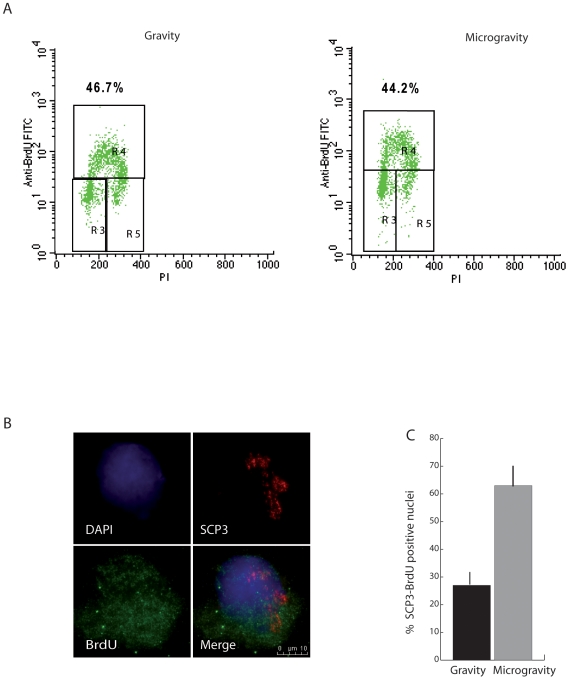
SM induces the last round of premeiotic DNA synthesis in Kit positive spermatogonia. A) Kit positive spermatogonia from 7 dpp mice were cultured for 24 h under gravity and SM conditions in the presence of BrdU and analysed by flow cytometry for BrdU incorporation and propidium iodide labeling. The upper quadrants show the limits for replicating cells. Percentages of cells in the different states are indicated. BrdU, bromodeoxyuridine. B) Representative immunofluorescence images of Scp3-positive (red) and BrdU-stained (green) premeiotic S-phase nuclei (preleptotene). Scale bar, 10 µm. C) Histogram showing the percentage of double positive cells respect to total BrdU stained nuclei after 24 of culture under gravity or SM condition. The values are means ± SD of three separate experiments. P≤0.05.

Kit-positive spermatogonia were cultured for 24 h under unit gravity or SM conditions in the presence of BrdU. Cells were harvested and BrdU positive cells were analyzed by flow cytometry for quantitation of DNA synthesis. As shown in Fig. 4A, the percentage of spermatogonia found in S phase did not change under microgravity. However, to ascertain whether a modification in DNA synthesis occurred selectively in specific cellular stages in the two culture conditions, nuclear spreads were simultaneously immunostained with anti-BrdU and anti-Scp3 antibodies. We observed an increase of about 2-fold in double positive cells (58.5% ±5) in RCCS culture with respect to cultures at unit gravity (26.6% ±3), as reported in Fig. 4B, C. BrdU labeling appeared to be uniform in nuclear spreads, rather than dotted or sparse, suggesting that it actually represents replicative DNA synthesis, rather than DNA repair events (Fig. 4B). Since most of the Scp3/BrdU positive nuclei (80%) found under SM conditions were at the preleptotene stage (Fig. 4B), we can conclude that microgravity stimulates the last round of pre-meiotic DNA synthesis, a prerequisite for the entry into meiosis.

**Figure 5:**
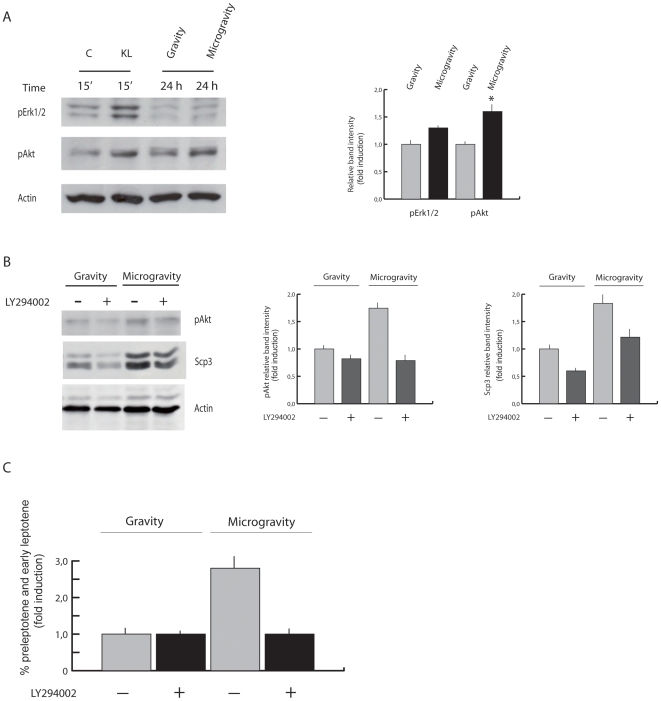
Akt activation by SM is required for induction of meiotic entry of Kit positive spermatogonia. A) Representative western blot of Erk1/2 and Akt phosphorylation state in Kit positive spermatogonia from 7dpp testes, cultured for 24 h in RCCS. Spermatogonia stimulated with KL for 15 min were used as positive control. Densitometric quantitation is shown in the right panel. B) Western blot analysis showing a decrease of phospho-Akt and Scp3 in spermatogonia cultured for 24 h under SM in the presence of 10 µM LY294002. Similar results were obtained in three separate experiments. Densitometric quantitation is shown in the right panels. C) Histograms representing the percentage of nuclei with a meiotic Scp3 organization (preleptotene and early leptotene morphology) after 24 h of culture at unit gravity and in SM, in the presence or absence of the Akt inhibitor LY294002. The values were obtained by counting 500 nuclei in each sample. Data are means ± SD of three independent experiments. P≤0.005.

We have recently demonstrated that meiotic entry of mitotic spermatogonia induced by ATRA and KL is mediated by the activation of PI3K and ERK pathways [10]. We investigated if the SM-induced differentiation of spermatogonia *in vitro* involved the activation of the same signalling pathways. Kit-positive spermatogonia were cultured in RCCS for 24 h, before cell protein extraction and analysis of activated Akt and Erk1/2. A reproducible increase in the phospho-Akt levels, and a slighter induction also of phospho-Erk1/2, were observed in cells cultured under SM with respect to cells cultured at unit gravity (Fig. 5A). Addition to the culture of the PI3K inhibitor LY294002 reverted the increase of the Scp3 protein levels induced by SM after 24 h of culture (Fig. 5B). LY294002 also abolished the increase in the number of preleptotene/early leptotene spermatocytes normally elicited by the RCCS conditions (fig. 5C), with no effects on cell survival, as determined by both the Trypan Blue exclusion test and TUNEL assay (data not shown). These results indicate the involvement of PI3K into the mitotic-meiotic transition of germ cells under SM condition.

## Discussion

In this work we show that mouse spermatogonia, cultured under SM conditions in RCCS, undergo a spontaneous differentiation and entry into the early prophase of the first meiotic division.

After 48 h of culture of isolated differentiating Kit-positive spermatogonia in RCCS, we found a dramatic increase in the number of meiotic nuclei, most of which were at the leptotene stage. A small number of zygotene nuclei were also present in the RCCS culture, which were never observed at unit gravity. The starting spermatogonia population consisted mostly of Scp3-negative germ cells (type A, intermediate and type B spermatogonia), and of a small number of preleplotene germ cells, which eventually shifted toward leptotene and zygotene stages under the RCCS conditions,. Due to the poor viability of Kit-positive spermatogonia after prolonged culture periods, we could not test whether more advanced meiotic stages can be reached after incubation times longer than 48 h. Further studies will be required to assess whether improvement of cell viability by addition of survival factors, hormones, or other pharmacological agents might extend meiotic progression of these cells under SM. The effect of SM on meiotic entry of differentiating spermatogonia was confirmed by the increased expression at the mRNA and/or protein level of genes important for commitment and entry into meiosis (such as *kit*, *scp3* and *stra8*) and of genes essential for early meiotic DNA recombination (such as *spo11*, *scp3* and *scp1*).

The effect of microgravity on spermatogonia differentiation was not exerted only on Kit-positive cells, but it was also evident on earlier differentiative stages of mitotic germ cells. In fact, when Kit-negative spermatogonia (obtained from 4dpp testes, after removal of Kit positive cells) were cultured for 24 h in RCCS, they underwent differentiation, as evaluated by increased expression of genes important for commitment of mitotic germ cells to the meiotic programm (Kit and Stra8), even though they failed to organize Scp3 onto chromosomes. However when total germ cell population from 4dpp testes, which includes Kit positive cell contaminant, were cultured in RCCS, they progressed to early meiotic stages, as evaluated by induction of Scp3 expression and its organization in preleptotene-like figures, which were never observed at unit gravity. This result strongly suggest that SM affects also the minority of Kit positive cells included in this population and confirm the previous data obtained from Kit-positive cells from testes of 7 dpp mice. However these cells were not able to progress towards leptotene stage compared to the Kit positive cells collected at 7dpp. These observations indicate that, in order to start a correct meiotic program, germ cells have to accomplish the differentiative events occurring during the Kit-positive stages of cell divisions, as discussed previously [10], [26]. The present data indicate that SM acts as an inducer or at least, an accelerator, of cell differentiation in post-natal mitotic germ cells, both at the Kit-negative and Kit–positive stages.

We found that SM had no apparent effect on total BrdU incorporation in Kit-positive spermatogonia cultured for 24 h. However, Scp3 co-staining revealed that RCCS conditions stimulated DNA synthesis selectively in preleptotene spermatocytes. This observation is in line with our results showing acceleration of meiotic entry after 48 h of RCCS culture, and suggests that SM induces progression toward meiosis by stimulating the last round of pre-meiotic DNA synthesis in Kit-positive spermatogonia.

The mechanisms underlying the positive effect of microgravity on germ cell differentiation are unknown. In an attempt to address this issue, we investigated whether PI3K and ERK pathways, known to mediate meiotic entry of spermatogonia induced by ATRA and KL [10], are also affected by the RCCS condition.

We found that after 24 h of culture a sustained activation of Akt accompanies the spontaneous progression toward the meiotic prophase of Kit-positive spermatogonia cultured under RCCS conditions. Erk1/2 activation after 24 h is less evident, but we cannot exclude that it occurred at earlier culture times. Thus, SM stimulates signalling pathways that physiologically mediate the transition from the mitotic to the meiotic cell cycle in Kit-positive spermatogonia. Addition of the PI3K inhibitor LY294002 abolished the increase in Scp3 expression and meiotic entry induced by SM. This observation suggests that induction of Scp3 expression under the RCCS conditions might be physiologically regulated by PI3K activity. This signalling pathway might be affected by the altered biomechanical forces imposed to the cells cultured in RCCS, including hydrostatic forces, circumferential stress, shear stress, metabolic changes, with consequent variations in the gene expression pattern of cultured mitotic germ cells. For instance, it is well known that shear stress induces Akt phosphorylation in endothelial cells [27]–[28]. Moreover, several studies have revealed that modification of biomechanical forces in *in vitro* culture systems are able to induce specific developmental programs in mesenchymal stem cells [29]–[30].

In conclusion, our results demonstrate that SM induces a spontaneous acceleration of differentiation in Kit-negative spermatogonia, and the meiotic entry of Kit-positive cells in the absence of any added growth factor or supporting somatic cells. Induction of meiotic entry is accompanied by stimulation of the last round of pre-meiotic DNA synthesis in mitotic Kit-positive cells and activation of the PI3K pathway.

Thus, SM might provide a simple, inexpensive and powerful tool to dissect at a molecular level the mechanisms underlying the switch from mitosis to meiosis during mammalian spermatogenesis.

## Materials and Methods

### Spermatogonia Isolation and Culture

This research, involving the use of laboratory mice, has been approved by the “Institutional Animal Care and Use” Committee of the University of Rome Tor Vergata. Mice were maintained and used in accordance with the guidelines issued by the Committee.

Enriched spermatogonia fraction was obtained from testes of immature 4 or 7 day-old Swiss CD-1 mice, as reported previously [10]. Briefly, after dissection of the albuginea membrane, testes were first digested with collagenase to remove interstitial cells and then with hyaluronidase and trypsin. The cell suspension was plated in Petri dishes for 4h in modified Eagle medium (MEM) supplemented with 10% fetal calf serum to promote adhesion of somatic cells. This pre-plating treatment allowed removal of most contaminating Sertoli and Leydig cells. Spermatogonia suspension was recovered and the purity was monitored morphologically after Giemsa staining, and immunocytochemically, as described previously [26]. From testes of 4dpp mice, a 50% enrichment of germ cells vs. somatic cells was obrtained. In this population, germ cells consisted mainly (90%) of Kit-negative spermatogonia, as monitored by FACS analysis. Kit receptor was immunostained with the APC- anti-mouse CD117 antibody (eBioscience). The homogeneity of the spermatogonial population from 7 day-old mice was about 85–90% including all stages: undifferentiated and differentiating (type A1–A4, intermediate, and type B) spermatogonia, 4.5% of preleptotene germ cells and rare leptotene spermatocytes. In this population 20%–25% of the cells were Kit-positive spermatogonia, as monitored by FACS analysis. Separation of Kit-positive spermatogonia was performed by using magnetic-activated cell sorting (MACS) with CD117 conjugated microbeads (Miltenyi Biotec, #130-091-224). For an accurate description of the purification protocol see http://www.miltenyibiotec.com/download/datasheets_en/264/DS130-091-224.pdf. Spermatogonia were cultured for 24 h or 48 h in MEM with 100 U/ml penicillin, 100 µg/ml streptomycin, 20 mM L-glutamine without serum supplementation, at unit gravity in conventional tissue culture dishes with PBS 0.5% agarose, or at microgravity in a rotary cell culture system (RCCS) (RCCS-4D; Synthecon Inc., El Rio, Houston, TX, USA). In the RCCS condition 4×10^6^ germ cells were seeded in disposable vessels with 10 ml MEM, using a rotation rate of 14 revolutions/min (RPM).

### Immunofluorescence and Western Blotting

For meiotic cell spreads, spermatogonia were prepared and stained essentially as described [10], [25]. Slides were washed twice in PBS, and incubated with anti-Scp3 rabbit polyclonal antibody (Novus NB 300-231, 1∶100) in blocking solution (10% serum from goat, 3% BSA, 0.05% Triton X-100 in PBS), overnight at 4°C. After washing, anti-rabbit FITC secondary antibody (Novus NB 730F, 1∶100) was added for 1 h at 37°C. The slides were washed and allowed to dry. Vectashield Mounting Medium with DAPI (Vector Laboratories) was added and the slides were analyzed using a Leica microscope.

For western blotting analysis, cells were lysed in 1% Triton X-100, 150 mM NaCl, 15 mM MgCl_2_, 15 mM EGTA, 10% Glycerol, 50 mM Hepes (pH 7.4) with protease inhibitors. Proteins were separated by SDS–10% polyacrylamide gel electrophoresis and transferred to nitrocellulose membrane (Amersham). The membrane was blocked in PBS–5% skim milk powder for 1 h. Incubation of the membrane with the primary antibody (1∶1000) was carried out at 4°C overnight in PBS–5% BSA and then with the appropriate horseradish peroxidase-conjugated secondary antibody (SantaCruz). Anti-Kit rabbit polyclonal (sc-6283), anti-phospho Erk1/2 mouse monoclonal antibody (sc-7383), anti-actin rabbit polyclonal (sc-7210), anti-Scp3 rabbit polyclonal was from Novus, anti-phospho Akt (Ser-473) from New England Biolabs and anti-Stra8 antibody from Abcam (ab 49602). The horseradish peroxidase conjugate was detected by chemiluminescence with an ECL Kit (Amersham) and autofluorography. Densitometry was performed using a Molecular Dynamics Densitometer and ImageQuant software. Protein values were normalized against tubulin or actin levels.

### Semi-Quantitative RT PCR Analysis

Total RNA from spermatogonia was isolated using Trizol Reagent (Life Technologies, USA). DNA was synthesized by SuperScript™ preamplification system for the first synthesis (Invitrogen) in a 12 µl reaction mix that contained 3 µg RNA and 1 µl Random primers. PCR was performed in a 50 µl reaction mix using 2.0 U *Taq* polymerase (Fisher), and 50 pmol primers for about 30 cycles (18–20 cycles for β-actin) with 1 µl cDNA as templates. The PCR products were examined using 1.5% agarose gel electrophoresis. Band density was evaluated by Molecular Dynamics Densitometer and ImageQuant software. RNA values were normalized against actin RNA levels.

Primer sequences for qRT-PCR were: *β-actin* forw 5′-GGTTCCGATGCCCTGAGGCTC and rev 5′-ACTTGCGGTGCACGATGGAGG; *spo11* forw 5′-CTGTTGGCCATGGTGAAGAGAGG and rev 5′- TCCTTGAATGTTAGTCGGCACAGC; scp3 forw 5′-ATG ATG GAA ACT CAG CAG CAA GAG A and rev 5′- TTG ACA CAA TCG TGG AGA GAA CAA C; *scp1* forw 5′-CAGGTATCAGAACTGTTGATCC; rev 5′-CTTCAGTGAGCTGAGAAATCGG; kit forw 5′- GCCACGTCTCAGCCATCTG, rev 5′- GTCGGGATCAATGCACGTCA,

### Bromodeoxyuridine/SCP3 Double Staining

According to the manual of BrdU labeling/detection kit (Roche, Nutley, NJ), 10 µM BrdU labeling medium was added to the spermatogonia cultures and allowed to incubate overnight at 32 °C under 5% CO_2_. Afterwards, germ cells were processed for both FACS analysis or spreads preparation. For FACS observation cells were washed two times in Washing buffer (PBS 0.5% Tween 20), fixed overnight with Ethanol 70% and stained with anti-Brdu (BD Biosciences, San Jose, CA, diluted 1∶50) according to the manufacturer's instruction. After two washing in PBS, 0.5% Tween 20 the cells were incubated with an anti-mouse FITC-conjugated secondary antibody (Alexa Fluor® 488 Dye 1∶350) at 4 °C for 45 min in the dark. Propidium iodide (1 µg/µl) was added before the FACS analysis. Spreads were obtained as previously described. After three washes with PBS, staining was performed with antibodies anti-BrdU (BD Biosciences, San Jose, CA, diluted 1∶40) and anti-Scp3 (Novus, 1∶100) in blocking solution containing DNaseI 1 U/ml ( Zymed) and 3 mM MgCl_2_, at 37 °C for 1 h. After washing, the cells were incubated with an anti-mouse FITC-conjugated secondary antibody for anti-BrdU and anti-rabbit secondary (Alexa Fluor® 568 1∶400) for Scp3. Cells were rinsed with PBS and incubated with 5 µg/ml DAPI for 10 min at room temperature followed by observation under a fluorescent microscope.

### Statistical Analysis

The Student t-test and ANOVA have been used to assess the significance, set at P<0.05. All experiments were performed from three to five times and at least in triplicate for each sample. Bars in the histograms represent standard deviations.
